# The Belgian Diabetes in Pregnancy Follow-Up Study (BEDIP-FUS): A Multi-Centric Prospective Cohort Study on the Long-Term Metabolic Risk across Different Degrees of Gestational Glucose Intolerance: Methodology and Design

**DOI:** 10.3390/jcm12031025

**Published:** 2023-01-28

**Authors:** Lore Raets, Kim Van Hoorenbeeck, Toon Maes, Chris Vercammen, Christophe De Block, Eveline Dirinck, Inge Van Pottelbergh, Katrien Wierckx, Annouschka Laenen, Annick Bogaerts, Chantal Mathieu, Katrien Benhalima

**Affiliations:** 1 Department of Endocrinology, University Hospital Gasthuisberg, KU Leuven, Herestraat 49, 3000 Leuven, Belgium; 2 Department of Pediatrics, Antwerp University Hospital, Drie Eikenstraat 655, 2650 Edegem, Belgium; 3 Department of Endocrinology, Imelda Ziekenhuis, Imeldalaan 9, 2820 Bonheiden, Belgium; 4 Department of Endocrinology-Diabetology-Metabolism, Antwerp University Hospital, Drie Eikenstraat 655, 2650 Edegem, Belgium; 5 Department of Endocrinology, OLV-Ziekenhuis Aalst-Asse-Ninove, Moorselbaan 164, 9300 Aalst, Belgium; 6 Center of Biostatics and Statistical Bioinformatics, KU Leuven, Kapucijnenvoer 35 bloc d-box 7001, 3000 Leuven, Belgium; 7 Department of Development & Regeneration, REALIFE Research Group KU Leuven, Herestraat 49, 3000 Leuven, Belgium

**Keywords:** gestational diabetes mellitus, type 2 diabetes mellitus, postpartum follow-up, glucose intolerance, glucose tolerance groups

## Abstract

The Belgian Diabetes in Pregnancy follow-up study (BEDIP-FUS) aims to investigate the impact of body mass index (BMI), adiposity and different degrees of glucose intolerance on the metabolic profile and future risk for type 2 diabetes (T2D) in women and offspring five years after delivery in the BEDIP study. The BEDIP study was a prospective cohort study to evaluate different screening strategies for gestational diabetes (GDM) based on the 2013 WHO criteria. The aim of the BEDIP-FUS is to recruit 375 women–offspring pairs, stratified according to three different subgroups based on the antenatal result of the glucose challenge test (GCT) and oral glucose tolerance test (OGTT) during the BEDIP pregnancy. The follow-up visit consists of a 75 g OGTT, anthropometric measurements and questionnaires for the mothers, and a fasting blood sample with anthropometric measurements for the child. Primary outcome for the mother is glucose intolerance defined by the American Diabetes Association criteria and for the offspring the BMI z-score. Recruitment began in January 2021. The BEDIP-FUS study will help to better individualize follow-up in women with different degrees of hyperglycemia in pregnancy and their offspring.

## 1. Introduction

Gestational diabetes mellitus (GDM) is described as diabetes first diagnosed during pregnancy provided that overt diabetes in early pregnancy has been excluded [1].Women with GDM have a seven-fold increased risk of developing type 2 diabetes (T2D) later in life compared to normal glucose tolerant (NGT) pregnant women [2,3]. After delivery, the underlying β-cell dysfunction and/or predisposing insulin resistance generally persist and are associated with the severity of glucose intolerance at the time of pregnancy [4]. Women with persistent glucose intolerance (impaired fasting glucose (IFG) and/or impaired glucose tolerance (IGT)) in early postpartum are a particularly high-risk group, with approximately one in two developing T2D within five years postpartum [5]. Women with GDM have a significantly higher risk of developing metabolic syndrome and cardiovascular disease, compared to normal glucose tolerant women [6,7]. Stratifying subgroups with a differential risk of developing an adverse cardiometabolic profile later in life offers a chance to better individualize the follow-up, management and prevention in this metabolic high-risk population. A Canadian postpartum follow-up study of different gestational glucose tolerance groups, based on the antenatal glucose challenge test (GCT) and 100 g oral glucose tolerance test (OGTT) using the National Diabetes Data Group criteria, showed that each degree of gestational glucose intolerance predicts distinct trajectories of β-cell function, insulin sensitivity and glycemia in the first three years postpartum which drive the differential risk of future T2D [4]. However, only limited data are available on the long-term risk of developing T2D in women with GDM diagnosed by the one-step approach and 2013 WHO criteria. Studies have shown that 30–50% of women with GDM develop T2D within the first 10 years after the index pregnancy before the introduction of the 2013 WHO screening [2]. An Irish study showed a rate of (pre)diabetes of 26% up to five years post-delivery in women with GDM diagnosed using the universal one-step approach and 2013 WHO criteria [8,9]. Additionally, we have demonstrated that gestational glucose tolerance worsens from normal to mildly abnormal (abnormal GCT but normal OGTT) to GDM in the BEDIP-N study based on a GCT ≥ 130 mg/dL and 75 g OGTT using the 2013 WHO criteria [10]. The future risk of developing T2D might gradually increase across these different glucose tolerance groups.

Additionally, screening tests such as the OGTT are time-consuming and uncomfortable, and they have limited reproducibility. Novel biomarkers that can adequately predict T2D after GDM are, therefore, needed. In diabetes, glycated CD59 is formed by non-enzymatic glycation which inactivates the complement inhibitor CD59. The research group of Prof J Halperin (Hematology lab Harvard) has demonstrated that by using a sensitive and specific enzyme-linked immunosorbent assay (ELISA) for glycated CD59 in blood, plasma glycated CD59 levels were significantly higher in individuals with T2D and independently predicted the response to the OGTT [11]. Glycated CD59 has also been shown to be a promising predictor for GDM and LGA [12]. We have previously shown that plasma glycated CD59 has a high negative predictive value in determining which women could avoid an OGTT in early postpartum [13]. We plan, therefore, to analyze glycated CD59 as a potential biomarker to predict the long-term metabolic risk for women with previous GDM and their offspring.

Offspring of women with GDM have an increased risk of becoming obese and develop T2D during their life [14,15]. Most human studies showing a relationship between in utero exposure to hyperglycemia and the occurrence of obesity and diabetes in the offspring have not investigated important confounders such as maternal and paternal obesity, socio-economic status and lifestyle behavior [16]. Increased maternal BMI is associated with increased childhood adiposity via mutual genetics, familial behavior, environmental aspects and the intrauterine setting [16]. Several studies have shown that adjusting for maternal BMI attenuates the associations between GDM and childhood obesity [17]. The follow-up of the HAPO study (HAPO-FUS) showed that among children of mothers with GDM based on the 2013 WHO criteria compared to those without GDM, the difference in childhood overweight and obesity defined by BMI was not statistically significant after adjustment for maternal BMI [18]. The HAPO-FUS study showed that, in children aged 10–14 years, there was an association between exposure to higher levels of glucose in utero and childhood glucose and insulin resistance, and this was independent of maternal and childhood BMI and family history of diabetes [19]. A Danish study evaluating the offspring of GDM mothers showed that, after correction for offspring BMI and maternal pre-pregnancy BMI, the offspring of women with GDM still had significantly higher fasting glucose, insulin resistance and waist-to-hip ratio compared to offspring of women without GDM [15]. By stratifying the results according to pre-pregnancy BMI, this study also suggests that hyperglycemia may be more significant in the absence of severe maternal adiposity [15].

More research is needed that determine the long-term metabolic risk of women and their offspring across different gestational glucose intolerance groups based on the 2013 WHO criteria. Therefore, we hypothesize in the BEDIP-FUS study that each degree of gestational glucose intolerance based on the antenatal GCT and OGTT with the 2013 WHO criteria predicts distinct postpartum trajectories of weight, β-cell function, insulin sensitivity and glycaemia in both women and offspring. In general, this research answers the need to better stratify women and offspring across different degrees of gestational glucose intolerance for their risk of developing an adverse cardiometabolic profile later in life. We hypothesize that the results of this research will help to optimize the follow-up, prevention and treatment of women with a previous history of different degrees of hyperglycemia in pregnancy.

## 2. Aim and Objectives of the BEDIP-FUS Study

We aim to investigate the impact of BMI, adiposity and different degrees of glucose intolerance on the metabolic profile and future risk for T2D in women and offspring across different gestational glucose tolerance groups on average five years after delivery in the BEDIP-N study. Additionally, we plan to evaluate the novel biomarker glycated CD59 to predict the long-term cardiometabolic profile in mothers and offspring.

To achieve this, the following specific objectives will be addressed in women and offspring across different gestational glucose tolerance groups on average five years after delivery:

- The prevalence of glucose intolerance (T2D and prediabetes);
- The prevalence of overweight and obesity;
- The degree of adiposity;
- The prevalence of metabolic syndrome;
- The degree of insulin resistance;
- The degree of β-cell dysfunction;
- Glycated CD59 as a predictor of metabolic dysfunction;
- Sex differences in metabolic profile in the offspring.

## 3. Methods and Analysis

### 3.1. Study Design and Setting

**Figure 1:**
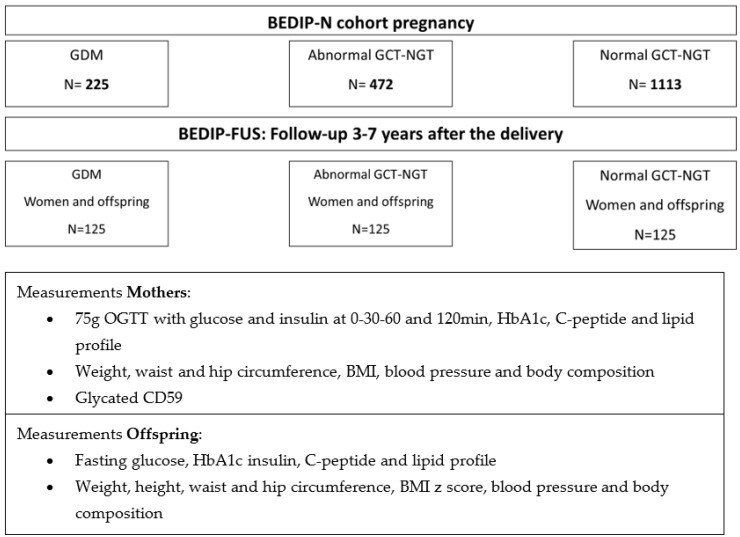
Study flowchart. BEDIP: Belgian Diabetes in Pregnancy; GDM: gestational diabetes mellitus; GCT: glucose challenge test; NGT: normal glucose tolerant (=normal OGTT); BEDIP-FUS: BEDIP follow-up; OGTT: oral glucose tolerance test; min: minutes; HbA1c: hemoglobin A1c; BMI: body mass index.

This is a Belgian multi-centric prospective follow-up study of the BEDIP-N cohort (Figure 1). The BEDIP-N study was a multicentric prospective cohort study to compare different screening strategies for GDM, which has been previously described in detail [10,20,21,22]. In this follow-up study, five of the original seven centers are participating (University hospital of Leuven (UZ Leuven), Antwerp University Hospital, OLVZ-Aalst, OLVZ-Asse and Imelda Bonheiden). UZ Leuven is the coordinating center. Of all participants in the BEDIP study, 95% consented to be contacted within 10 years after the study end to potentially participate in a follow-up study. Women who consented to be contacted, are invited by email, phone or letter to participate in the BEDIP-FUS study. Our study is registered in ClinicalTrials.gov as NCT04429958 since June 2020 and was approved by the Medical Ethical Committees of all participating centers.

### 3.2. Recruitment and Eligibility

We aim to recruit 375 women–offspring pairs with the five largest centers who participated in the initial BEDIP study, which is approximately 21% of the initial 1800 women who received both a GCT and 75 g OGTT in the BEDIP study. Women and offspring are recruited according to three different subgroups based on the antenatal result of the GCT and OGTT in the index pregnancy (a sample of 125 in each group): GDM group; NGT on the OGTT with an abnormal preceding GCT (abnormal GCT-NGT group); NGT on the OGTT with a normal preceding GCT (normal GCT-NGT group). Abnormal GCT is defined as glucose ≥ 130 mg/dL, as we have previously shown to have an acceptable sensitivity to screen for GDM in our population (20). Additional analyses will be performed on the different subgroups stratified by a GCT ≥ 140 mg/dL to evaluate whether this leads to more pronounced differences in β-cell function and insulin sensitivity across the different groups. GDM was defined by the 2013 WHO criteria. Recruitment began in January 2021 and will end around December 2023.

Eligibility criteria:

The inclusion criteria for the mother are:

- Age ≥ 18 years;
- Participated in the completed BEDIP-N study and received both the GCT and the OGTT during pregnancy;

The inclusion criterion for the offspring is:

- Born at the time of participation in the BEDIP-N study.

The inclusion criterion for the father is:

- Biological father of the child born during participation in the BEDIP-N study.

The exclusion criteria for the mother are:

- Current pregnancy;
- Current treatment that influences glycemic status, such as high dose corticoids or incretin mimetic medication;
- History of bariatric surgery or any gastrointestinal surgery that alters glucose absorption (Billroth II);
- Inability to complete a normal study visit (incompliance, psychiatric problems);
- Diagnosis of type 1 diabetes or the presence of autoimmune antibodies for type 1 diabetes.

The exclusion criteria for the offspring are:

- Current treatment that influences glycemic status, such as high dose corticoids;
- Inability to complete a normal study visit (incompliance, psychiatric problems);
- Diagnosis of type 1 diabetes or the presence of autoimmune antibodies for type 1 diabetes.

### 3.3. Study Visit

Women and their offspring born during the BEDIP-N study will receive one follow-up study visit three to seven years postpartum, which for the mother consists of a 75 g OGTT test, anthropometric measurements and questionnaires (about the mothers and their offspring) and for the child consists of fasting blood sampling and anthropometric measurements. Furthermore, baseline characteristics and data on medical and obstetric history are collected. The biological father of the child is also asked to complete a questionnaire on sociodemographic data and (family) history for cardiometabolic diseases.

#### 3.3.1. Lab Measurements

In women, after a 10 h overnight fast, hemoglobin A1c (HbA1c), C-peptide, and lipid profile (total cholesterol, triglycerides, high-density lipoprotein (HDL) and low-density lipoprotein (LDL) cholesterol) are measured, and a 2 h 75 g OGTT is performed, with measurements of glucose and insulin fasting, at 30 min, 60 min and 120 min. In offspring, fasting glycemia, HbA1c, insulin, C-peptide and lipid profile will be measured. Both in mothers and offspring, a plasma sample will be taken to allow for future analysis of plasma glycated CD59 by Harvard Prof. J Halperin’s lab. In addition, two extra plasma samples will be collected to allow for analysis of new (not yet defined) biomarkers in the future. In the long-term, blood samples will be stored at −80 °C in the UZ Leuven biobank.

Venous blood is collected by a nurse. For the 75 g OGTT, participants are asked to fast for at least 10 h and to not smoke prior to the test. During the test no physical activity is allowed. They are also instructed to only drink water but no other drinks with sugar or caffeine. The test starts with a fasting blood sample. After the fasting blood collection, women are instructed to consume a 75 g glucose beverage (Glucomedics^®^, Lambra, Madrid, Spain) in 5 min. After consumption of this beverage, separate blood collections at 30 min, 60 min and 120 min follow.

For the fasting blood test in the offspring, participants are asked to fast for at least 8 h. Prior to the blood sampling, they can only drink water.

All blood measurements will be centrally analyzed in the lab at UZ Leuven to assure uniformity of the analysis. Only the glucose analyses will be done locally to avoid any delay in diagnosing diabetes. The blood samples will be stored at the local center at −20 °C for a maximum of three months before shipment to UZ Leuven. Blood samples will then be stored at −80 °C in the UZ Leuven biobank. Coefficients of variance are 1% for glucose, 6% for insulin, 2% for lipids, 2% for HbA1c and 8% for C-peptide in the UZ Leuven lab.

#### 3.3.2. Clinical Measurements

Blood pressure and heart rate are measured using a calibrated Omron automatic blood pressure monitor. Height is measured to the nearest 0.5 cm using a calibrated wall-mounted stadiometer. Weight is measured to the nearest 0.1 Kg using a calibrated portable digital scale. BMI is calculated as kg/m^2^ for women and as BMI z-score for offspring [23]. Waist circumference is measured to the nearest 0.1 cm by applying the tape directly on the skin, horizontally at the lateral level midway between the iliac crest and the lowest lateral portion of the rib cage. Hip circumference is measured to the nearest 0.1 cm over the widest part of the gluteal region. Offspring are weighed without shoes and lightly dressed. Body fat is measured by bioelectrical impedance analysis (Bodystat 1500, Bodystat Limited, Douglas, Isle of Man, Great Britain). Skinfolds are measured at three different sites (triceps, subscapular, suprailiac, measured twice at each site with calibrated calipers (Harpenden, Baty International, West Sussex, Great Britain) to the nearest 0.1 mm (if results differed by >1.0 mm, they were measured a third time)) [18]. Means of the two skinfold measurements, or the two closest measurements if three measurements were made, are used for analysis. All participating centers received specific training for the skinfold measurements in order perform these measurements correctly. In addition, instructional videos were made for skinfold measurements to refresh training if necessary.

All anthropometric measurements are taken twice, and if the differences exceed 0.5 cm or 0.5 kg for the anthropometric measurements, or 5 mmHg for blood pressure measurements, a third measurement is taken. In all analyses, the mean value of the measurements is used. The anthropometric measurements are performed by a trained nurse, physician or clinical trial assistant.

#### 3.3.3. Self-Administered Questionnaires

The following self-administered questionnaires are used:

For the mother:

- Self-designed questionnaire on general habits (including smoking and alcohol habits) and socioeconomic factors, as used in the BEDIP-N study [22];
- The Frequency Food Questionnaire (FFQ) validated for the Belgian population [24] to assess food and nutrition intake. This is a questionnaire that contains questions on portion and frequency of food and beverage consumption;
- The international questionnaire on physical activity (IPAQ), validated for use in the Belgian population and as used in the BEDIP-N study for the mothers [22,25]. This is a questionnaire measuring different areas of physical activity such as job-related physical activity, transportation, housework and caring for family, recreation and time spent sitting. We added a question on time watching television or playing computer games to better assess sedentary behavior;
- The validated 20-item Center for Epidemiologic Studies Depression Scale (CES-D) questionnaire [26] to assess symptoms of depression in the past 7 days;
- A self-designed questionnaire on breastfeeding and contraception as used in the BEDIP-N study [22];
- The validated questionnaire on general health (SF-36) [22,27] to assess quality of life;
- The validated Diabetes Risk Perception questionnaire [28];
- The validated Treatment Self-Regulation Questionnaire (TSRQ) to evaluate motivation for lifestyle change [29];
- The validated short version STAI-6 to measure anxiety [30];
- The validated Pittsburgh Sleep Quality Index to evaluate sleep quality as a risk factor for the development of glucose intolerance [31].

For the offspring (completed by the mothers):

- The validated FFQ for children [32] to evaluate food and nutrition intake;
- The validated Dutch Physical Activity Questionnaires for Children [33];
- The child health questionnaire—parent form (CHQ-PF50) validated to assess health-related quality of life for children [34].

For the father:

- Self-designed questionnaire on general habits (including smoking and alcohol habits) and socioeconomic factors as used in the BEDIP-N study [22] including data on paternal weight and BMI over time.

### 3.4. Outcomes of the Study

#### 3.4.1. Primary outcome

For mothers: A disorder of glucose metabolism: T2D defined by the 75 g OGTT and/or HbA1c, or prediabetes defined as IFG and/or IGT by the American Diabetes Association (ADA) criteria [1].

For offspring: BMI z-score as a continuous variable [23].

#### 3.4.2. Secondary Outcomes

Secondary outcomes for mothers:

- BMI as continuous variable and as overweight: BMI ≥ 25 kg/m^2^ and obesity BMI ≥ 30 kg/m^2^;
- Metabolic syndrome based on the WHO criteria [35];
- Insulin sensitivity measured by the Matsuda insulin sensitivity index, a well-established measure of whole-body insulin sensitivity [36], and the reciprocal of the homeostasis model assessment of insulin resistance (1/HOMA-IR) (largely hepatic measure of insulin sensitivity) [37];
- Beta-cell function by the HOMA-B index and the insulinogenic index divided by HOMA-IR [38], the insulin-secretion sensitivity-2 index [39], and the Stumvoll index [40]. All these measures have been validated for use in women with GDM and have been used in the BEDIP-N study [22];
- Adiposity (as a continuous variable) measured by the bioelectrical impedance analysis (BIA), and measured by skinfolds [41];
- Plasma glycated CD59 as a predictor of metabolic risk [11,13].

Secondary outcomes for offspring:

- Overweight and obesity defined by BMI z-score according to the WHO guidelines (resp. score >1 and >2) [23];
- Prediabetes and diabetes based on the fasting plasma glucose defined by the ADA criteria [1];
- Metabolic syndrome based on the WHO criteria [35];
- Insulin sensitivity by 1/HOMA-IR [37];
- Beta-cell function by the HOMA-B index [22];
- Adiposity measured by BIA and measured by skin folds [41];
- Weight and growth trajectory;
- Plasma glycated CD59 as a predictor of metabolic risk [11];
- Sex differences in metabolic profile in the offspring.

### 3.5. Collection of Data from the Medical Electronical Records

For the mothers, we are collecting data concerning previous pregnancies other than the one during the BEDIP-N study. The following data are collected: pre-pregnancy weight, weight in early pregnancy, weight at delivery, pregnancy outcomes, gestational weight gain, parity, medical history, current treatment, history of metabolic syndrome, history of (pre)diabetes, diabetes related complications (retinopathy, microalbuminuria, nephropathy, neuropathy), cardiovascular diseases (heart, cerebral and peripheral arterial disease).

For the offspring, we will collect the following data from their medical records: medical history, current treatment, history of (pre)diabetes, information on school attendance (which school, which school year). Data on growth and weight trajectory, and data on the development of the child, will be collected through the databases used by the Flemish health services for the normal follow-up of children.

### 3.6. Power Calculation and Statistical Analyses

#### 3.6.1. Sample Size

Since there are currently no data on the long-term metabolic risk of lesser degrees of hyperglycemia than GDM based on the GCT and 75 g OGTT using the currently recommended 2013 WHO criteria, we based our power calculation on the Canadian study by Ravi Retnakaran et al. in which women received the GCT and 100 g OGTT using the National Diabetes Data Group criteria [4]. The distribution of the patients over the groups was based on the BEDIP-N study as previously published [10]. The power calculation is performed for the primary outcome for mothers: glucose intolerance after on average five years. We aim to compare glucose intolerance as a binary outcome variable between the GDM group (3), where a 26% prevalence is assumed, and (1) normal GCT-NGT group and (2) abnormal GCT-NGT group, with an assumed prevalence of 4% and 12%, respectively, with 125 subjects in each group. Considering a chi-square test, the power for the comparisons of the primary outcome at a 5% significance level, is 99% (1 vs. 3) and 81% (2 vs. 3), respectively.

#### 3.6.2. Statistical Analyses

Differences between groups will be analyzed using linear models in the case of continuous outcomes and logistic regression models in the case of binary outcomes. Results will be presented as mean differences or odds ratios, respectively, with 95% confidence intervals.

Analyses will be performed separately and analogously for comparing (1) GDM versus non-GDM, and (2) the three gestational glucose tolerance groups. In the analyses on offspring, interaction effects between the group and the gender of the offspring will be analyzed. Such an interaction effect would mean that the differences in outcome between the groups is different for males and females. In the case of a group by gender interaction effect, results will be presented by gender. In the absence of such an interaction, the main effects for the group will be presented.

Multivariable models will be used to correct for possible confounders. Correction for confounders will be performed in a stepwise fashion. For maternal outcomes, covariate adjustment will be performed for time since pregnancy in a first step. Maternal variables during pregnancy will be added as confounders in a second step, and maternal variables during follow-up will be further added in a third step. For outcomes in offspring, covariate adjustment will be performed for the child’s age and center in a first step. Maternal variables during pregnancy will be added as confounders in a second step, and offspring variables during follow-up will be further added in a third step.

Maternal characteristics during pregnancy include variables such as age, pre-pregnancy BMI, blood pressure, and smoking. Follow-up characteristics include variables such as mothers’ age, BMI measured during the OGTT and smoking for maternal outcomes, and variables such as children’s age and BMI at the time of the clinical examination for analyses on offspring outcomes. The confounders included in each analysis will depend on the concrete outcome that is being analyzed. Correction is indicated if the confounder is known or suspected to be associated with the predictor of interest (group) as well as the outcome. Confounders will be determined a priori based on expertise and literature. If the number of indicated confounders is too large in relation to the sample size, further selection will be based on univariable analyses on the association between the confounder and the group.

### 3.7. Quality Control Procedures

Participating sites started recruitment after a first initiation visit. During the course of the study, the study monitor will perform periodic monitoring visits to ensure that the protocol is being followed. We plan at least a yearly monitoring visit for all sites. The coordinators of the study may review source documents to confirm that the data recorded on CRF pages are accurate. Collected data are recorded in the Good Clinical Practice (GCP) compliant Electronic Data Capture (EDC) platform ‘Research Data Capturing’ (REDCap). The monitor can assure data quality in the eCRF by sending queries to the local coordinators, in addition to on-site monitoring every four to six months. Our study has provided detailed standard operating procedures describing data collection during the study visit and blood sample collection.

## 4. Discussion

The BEDIP-FUS study will contribute to the push for more studies to determine the long-term metabolic risk of women across different gestational glucose tolerance groups based on the 2013 WHO criteria. Applying these 2013 WHO criteria for GDM could result in a greater percentage of women being diagnosed with mild forms of GDM, which might lead to a lower portion at risk for postpartum glucose intolerance. Studies are needed to determine the long-term metabolic risk of women across different gestational glucose tolerance groups based on the 2013 WHO criteria. Prospective studies are also needed to evaluate novel biomarkers, such as glycated CD59, to predict the long-term metabolic risk in woman and offspring. These results might help us to find a more accurate and patient-friendly way of screening for glucose intolerance, and may help to stratify women and offspring with a differential risk of developing an adverse cardiometabolic profile later in life.

To the best of our knowledge, we are the first study to investigate the impact of BMI, adiposity and different degrees of glucose intolerance on the metabolic profile and future risk for type 2 diabetes (T2D) in women and offspring across different gestational glucose tolerance groups in pregnancy based on the 2013 WHO criteria. In addition, we will also investigate whether different glucose tolerance groups measured with a 50 g GCT (cut-off: 130 mg/dL) followed by a 2 h 75 g OGTT identifies distinct trajectories of β-cell function, insulin sensitivity, and glycaemia approximately five years postpartum.

Studies provide no evidence yet that treating GDM in pregnancy can reduce the future risk for obesity and glucose intolerance in mothers and offspring. Reduction of macrosomia at birth was seen in women with GDM who received intensified glucose-lowering treatment, but follow-up studies of offspring showed no decline in childhood obesity or metabolic dysfunction in these children at the age of 4–10 years [42,43]. However, in an American study, sex-specific differences were seen, with a decreased frequency of impaired fasting glucose, lower fasting glucose, and lower insulin resistance in female offspring but not in male offspring [43]. Differing sensitivities to hyperglycemia in utero may result in long-term sex-specific programming. Additional studies are needed to understand the separate role and the combined potential superimposing effect of maternal hyperglycemia, maternal obesity during pregnancy and paternal BMI on the metabolic health of offspring. Moreover, data on the metabolic profile of offspring of women diagnosed with GDM according to the 2013 WHO criteria are scarce. Studies should also investigate sex differences in metabolic profiles in the offspring. With the BEDIP-N follow-up study we aim, therefore, to also evaluate potential sex differences in long-term metabolic risk across different gestational glucose tolerance groups in pregnancy.

A major strength of our study is that all women received both a GCT as well as an OGTT during pregnancy, allowing investigation of the impact of different glucose tolerance groups during pregnancy on the long-term glucometabolic risk of women and their offspring. We also have a large dataset on pregnancy and delivery outcomes for both mother and offspring. A limitation of our study is that our cohort mainly consists of Caucasian women.

## 5. Conclusions

Our study may help individualize the follow-up and intervention strategies in women and offspring with a history of GDM and different degrees of glucose intolerance based on the 2013 WHO criteria.

## Data Availability

This is the publication of the study protocol of an ongoing study. Data of the study are not available for publication, since recruitment is ongoing. Data sharing is not applicable to this article.
